# Effect of *Toxoplasma gondii* on Ram Sperm Quality after Experimental Infection

**DOI:** 10.3390/pathogens9121004

**Published:** 2020-11-30

**Authors:** Theofanis Fais, Nektarios Giadinis, Elias Papadopoulos, Georgia Brellou, Alexandros Theodoridis, Radu Blaga, Delphine Le Roux, Dimitra Bitchava, Aikaterini Ntemka, Constantin Boscos, Ioannis Tsakmakidis

**Affiliations:** 1 Unit of Biotechnology of Reproduction, Farm Animals Clinic, School of Veterinary Medicine, Faculty of Health Sciences, Aristotle University of Thessaloniki, 54627 Thessaloniki, Greece; theofanf@vet.auth.gr (T.F.); ntemka@vet.auth.gr (A.N.); pboscos@vet.auth.gr (C.B.); 2 Farm Animals Clinic, School of Veterinary Medicine, Faculty of Health Sciences, Aristotle University of Thessaloniki, 54627 Thessaloniki, Greece; ngiadini@vet.auth.gr; 3 Laboratory of Parasitology and Parasitic Diseases, School of Veterinary Medicine, Faculty of Health Sciences, Aristotle University of Thessaloniki, 54124 Thessaloniki, Greece; eliaspap@vet.auth.gr; 4 Laboratory of Pathology, School of Veterinary Medicine, Faculty of Health Sciences, Aristotle University of Thessaloniki, 54627 Thessaloniki, Greece; mprellou@vet.auth.gr; 5 Laboratory of Animal Production Economics, School of Veterinary Medicine, Faculty of Health Sciences, Aristotle University of Thessaloniki, 54124 Thessaloniki, Greece; alextheod@vet.auth.gr; 6 UMR BIPAR, Ecole Nationale Vétérinaire d’Alfort, ANSES, Animal Health Laboratory, National Reference Laboratory for Foodborne Parasites, Université Paris-Est, INRA, Paris, 14 rue Pierre et Marie Curie, 94700 Maisons-Alfort, France; radu.blaga@vet-alfort.fr (R.B.); delphine.le-roux@vet-alfort.fr (D.L.R.); 7 Veterinary Laboratories Vet in Progress plus, 2 Kritis and Agias Theklas, 15343 Athens, Greece; information@vetinprogress.gr

**Keywords:** *Toxoplasma gondii*, ram, sperm, testis, epididymis, puberty, experimental

## Abstract

The aim of this study was to investigate the effect of experimental *Toxoplasma gondii* infection on ram sperm quality. Five months old, pre-pubertal, rams were divided into four groups (*n* = 8 per group). Group A was the control group; the remaining animals received *per os* (p.o.) 5000 oocysts per ram. Group B did not receive treatment post-infection (p.i.). Group C received sulphadimidine (intermuscular injection (i.m.) 33 mg/kg for eight days; every 48 h) two months p.i. and Group D received the same drug twice (24 h p.i. and two months later). Blood samples were collected every 15 days to detect serum immunoglobulin G (IgG). Epididymal sperm samples were analyzed for concentration, kinetics, morphology/viability, functional membrane integrity, DNA integrity, and the presence of parasite DNA. Histopathological examination was performed on the testes. The IgG titres in infected groups raised two weeks p.i. and remained high for four months. Higher values were noticed in viability and functional membrane integrity in positive spermatozoa in the control group compared to other groups, level of significance *p* < 0.05. Abnormal sperm was higher in groups C and D vs. A and C vs. B (*p* < 0.05). *T. gondii* DNA was detected in three sperm samples of the infected rams (12.5%). Histopathology revealed similar findings with little variation among all infected groups, characterized mostly by increased interstitial connective tissue, non-purulent inflammation, and presence of seminiferous tubules with spermatogenic cell depletion, which increased gradually from D to C and B groups. In conclusion *Toxoplasmosis* in pre-pubertal age negatively affected mature ram sperm quality, while sulphadimidine administration failed to alter this.

## 1. Introduction

*Toxoplasma gondii* is an intracellular protozoan parasite with a complex life cycle involving cat and other Felidae family members as definitive hosts, while virtually all-warm-blooded animals, including humans, can act as intermediate hosts [1].

*T. gondii* infection in meat-producing animals is mainly considered from a public health point of view since humans can be infected by ingesting raw or undercooked meat containing the parasite stage of bradyzoites within tissue cysts [2]. However, this infection is at the origin of economic losses in animal husbandry due to various reproductive disorders such as early embryonic death, abortion, stillbirth, and neonatal death, especially in small ruminants [3,4].

Whether these reproductive disorders are due to a *T. gondii* external contamination, by ingesting oocysts, or by infected semen are not well understood. All we know is that the presence of *T. gondii* has been already detected in the male genital system and the semen of experimentally infected boars [5], rams [6], and bucks [7], while rams infected subcutaneously with *Toxoplasma* cysts produced infected sperm 14 to 26 days post-infection (p.i.) [8]. Furthermore, DNA of the parasite was detected in fresh and frozen ram semen from commercial artificial insemination centers and sheep farms in Brazil [9,10] and Tunisia [11]. The transmission of *T. gondii* through natural mating from experimentally infected rams [12], and male goats [13] to females, with consequent vertical transmission to their offspring, as well through insemination with fresh ram semen experimentally contaminated with *T. gondii* tachyzoites [14], has also been demonstrated. In addition, it has been shown that ram sperm freezing protocol cannot prevent *T. gondii* transmission by laparoscopic artificial insemination [15].

On the other hand, these reproductive disorders may be due to a faulty sperm that affects fertility. Semen characteristics such as concentration, viability, morphology, motility, functional membrane integrity, and sperm DNA integrity determine sperm quality and fertilizing ability [16]. Research results in humans and animals pointed out that adverse effects on the male genital system during the pre-pubertal period negatively affect its later reproductive capacity [17]. However, studies in various species provided different results of *T. gondii* effects on sperm quality and male reproductive histopathology. Terpsidis et al. [18] reported that toxoplasmosis affected male rat semen variables, but no pathological lesions were detected in testes. However, Arantes et al. [19] found alterations in dog testis and epididymides, which were attributed to toxoplasmosis infection. On the other hand, De Moura et al. [20], and Lopes et al. [21], found no or minimal sperm alterations.

Therefore, our first goal was to investigate the effect of the protozoan *T. gondii* on mature ram sperm quality after the experimental infection of premature males since there are no such data in the literature. Moreover, the treatment of toxoplasmosis in ewes involves the administration of monensin [22], sulphadimidine [23,24], or sulphamezathine and pyrimethamine in combination [25]. Since there are no reports about the effect of sulphadimidine administration on *T. gondii* contaminated rams, the second goal of the present study was to explore the possible efficacy of sulphadimidine treatment in *T. gondii* experimentally infected premature rams.

## 2. Results

### 2.1. Clinical Findings

The uninfected animals of group A did not present any clinical signs. Anorexia and an increase in respiratory rate were observed in all infected animals, from the 4th to the 10th-day p.i. The most notable clinical sign was hyperthermia starting on the 4th day p.i., reaching in some animals of all infected groups a peak on the 6th-day p.i. (42.3 °C). Apathy was also observed in animals that appeared with the highest body temperature. No apparent clinical symptoms were observed in infected animals after the 12th-day p.i. No clinical differences were observed between B (untreated), C, and D (treated) groups.

### 2.2. Serology

**Figure 1:**
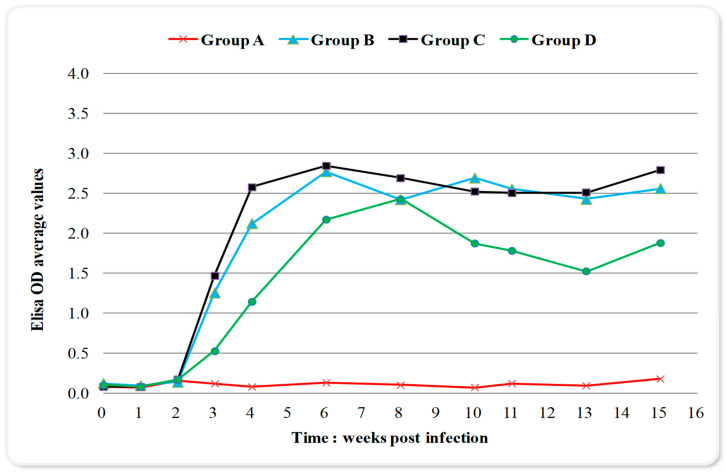
Changes in serum IgG titers (expressed as optical density (OD) values; positivity threshold 0.595) over time during the experiment in control and infected with *Toxoplasma gondii* groups. Group A control, group B infected, group C infected and treated with sulphadimidine on week eight p.i., group D infected and treated with sulphadimidine twice (24 h p.i. and in week eight). The seropositivity started increasing after the 2nd-week p.i. and remained high until the 15th-week. In group D, a delay of one week in immune response and lower values of seropositivity were noticed compared to groups B and C.

*Toxoplasma* infection caused a late immunological response to all inoculated groups detected by enzyme-linked immunosorbent assay (ELISA) (Figure 1). The results were expressed as optical density (OD) values while an antibody titer of 0.595 or above was considered positive Changes in blood serum IgG antibodies (Abs) titers started increasing after the 2nd-week p.i. and remained high from week six until week 15 p.i. (Figure 1); with no significant differences over time (the differences of the mean values of IgG titers between weeks are presented in Table 1). The maximum value of 3.654 OD was recorded in week eight p.i. in group C (Figure 1). In group D, which received sulphadimidine twice (24 h and week eight p.i.), a one-week delay in immune response and significantly lower mean values of seropositivity were observed compared to groups B and C, almost over the entire experimental time (Figure 1, Table 2). The IgG titer mean values for group B and C showed a peak on week six p.i., compared to a one-week delay for group D (Figure 1). When examining IgG titers across treatment groups (main effects for treatment), results indicated that group D had significantly lower values in immune response compared to other infected groups (Table 3). Group C, which received sulphadimidine on week eight p.i., had no significant differences in immune response compared to untreated group B in total (Table 3), but also at any time-point (Figure 1, Table 2). An increase in antibody titers after week 13 was observed in all infected groups (Figure 1). The results of serum samples from control group A were seronegative at all measurement time points.

### 2.3. Molecular Analysis

*Toxoplasma gondii* DNA was detected by polymerase chain reaction (PCR) in three sperm samples of the infected rams (12.5%), one animal per infected group (B, C, and D). No trace of the parasite DNA was present in control samples.

### 2.4. Sperm Quality Parameters

#### 2.4.1. Concentration

Table 4 presents the results, which revealed higher concentration values in the control (A) group compared to infected groups as follows: A>B>D>C; but no significant difference between any groups was observed (*p* > 0.05).

#### 2.4.2. Viability

Viability was significantly higher in control group A compared to all infected groups (*p* < 0.05) (Table 4).

#### 2.4.3. Sperm Morphology

The statistical analysis of the results (Table 4) showed significantly higher total morphological abnormalities in group C compared to A and B (*p* < 0.05), as well as in group D compared to A. Regarding the individual sperm abnormalities, significantly higher values were observed in head defects of C compared to A and D groups and in tail abnormalities of C compared to A and B groups (*p* < 0.05). No significant differences were noticed between groups concerning the midpiece abnormalities and the presence of cytoplasmic droplets (*p* > 0.05).

#### 2.4.4. Motility and Kinetics

Computer-assisted sperm analysis (CASA) results are presented in Table 5. Values of Curvilinear Velocity (VCL), Straight Line Velocity (VSL), Average Path Velocity (VAP), Amplitude of Lateral Head displacement (ALH), and the percentages of total motility, progressive, rapid, medium, slow-moving, immotile, and hyperactivated spermatozoa had no significant differences between groups (*p* > 0.05). Analysis of the results showed significantly higher values of Beat-Cross Frequency (BCF) in group B compared to group C, Linearity (LIN) in groups B and C compared to group A, and Straightness (STR) in group C compared to group A (*p* < 0.05).

#### 2.4.5. Hypo-Osmotic Swelling Test (HOS-Test), Sperm Membrane Biochemical Functionality

The estimation of functional integrity of sperm plasma membranes by hypo-osmotic swelling test (HOST) revealed (Table 4) a significantly higher percentage of HOST-positive spermatozoa (%) in control compared to all the remaining groups (*p* < 0.05).

#### 2.4.6. Sperm DNA Integrity

The results of an Acridine Orange Test (AOT) revealed (Table 4) that spermatozoa nuclear chromatin fragmentation varied from 0% to 0.6%, except one animal of group C with 4.2% and one animal of group D with 1% DNA fragmentation. Statistical analysis showed no significant differences between groups (*p* > 0.05).

### 2.5. Histopathological Findings

**Figure 2:**
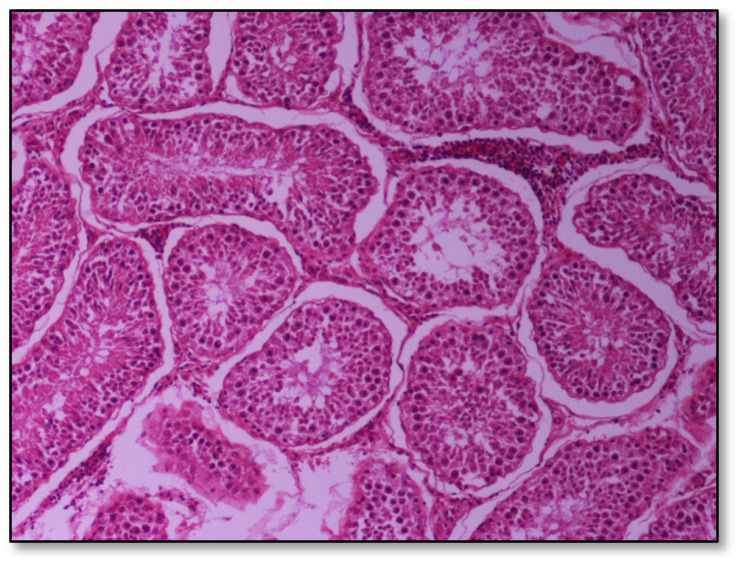
Group D, one large and two smaller foci of non-purulent (lymphocytic, plasmacytic) infiltration of the interstitial tissue. Note the germ cell exfoliation and absence mostly of late/elongated spermatids in the tubular lumen. When observed, they presented with morphological abnormalities, e.g., coalescence; original magnification ×10.

**Figure 3:**
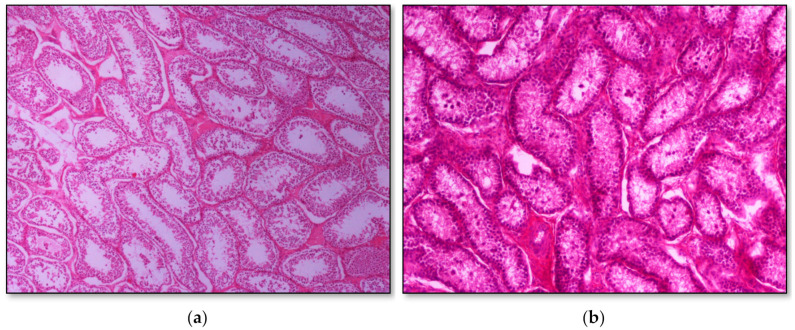
Seminiferous tubule depletion. (**a**) Group C, focal increase of interstitial connective tissue separates further adjacent tubules; original magnification ×4; (**b**) Group B, multifocal increase of interstitial connective tissue separates further adjacent tubules; original magnification ×10.

**Figure 4:**
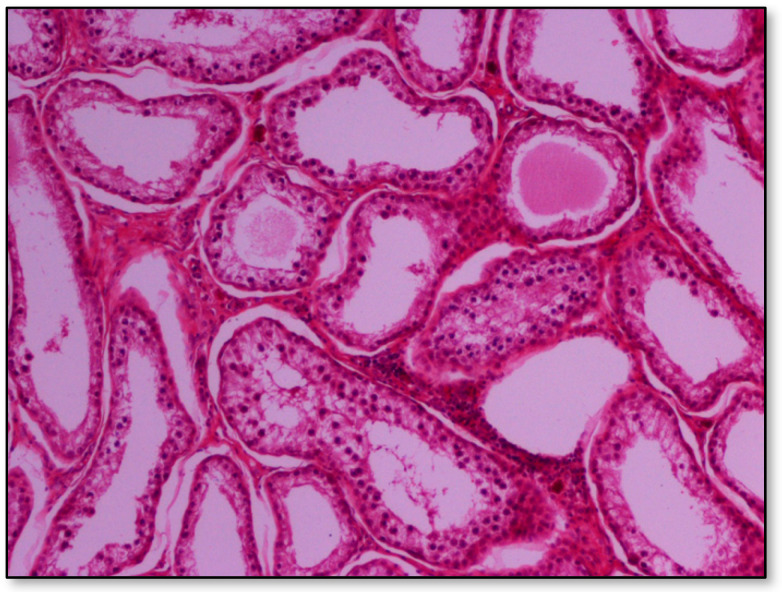
Group B marked depletion (degeneration) and distention of seminiferous tubules. A few sertoli cells remain in the majority of tubules; original magnification ×10.

**Figure 5:**
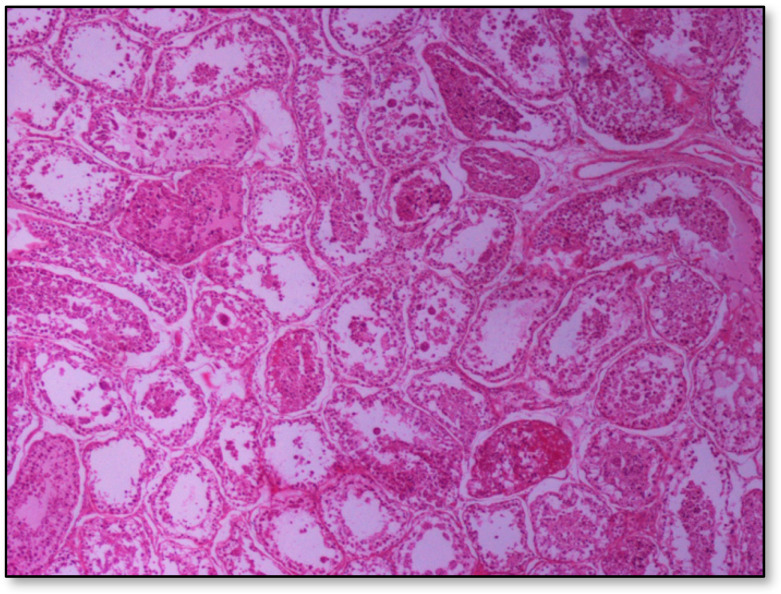
Group C, in addition to the depletion in the absence of spermatids, multiple tubules show amorphous eosinophilic material and/or intraluminal multinucleated giant cells; original magnification ×4.

Histopathology of testes revealed similar findings with little variation among all infected groups, characterized mostly by, (i) non-purulent inflammation (Figure 2), (ii) increased interstitial connective tissue (Figure 3a,b), and (iii) presence of seminiferous tubules with spermatogenic cell depletion, which increased gradually from D to C and B group (Figure 2, Figure 3a,b, Figure 4 and Figure 5).

## 3. Discussion

The sexual transmission of *T. gondii* in small ruminants is still a subject to debate in terms of epidemiological importance, with pros [7,12,26] and cons [8]. Our study was intended to add new information concerning *T. gondii* effect on mature ram sperm quality and *T. gondii* putative involvement in reproductive disorders within small ruminant husbandry.

Two hundred oocysts are the threshold value for the induction of *Toxoplasma* infection in sheep [27]. In our experiments, we used a dose of 5 × 10^3^ mature oocysts of the parasite *T. gondii* capable of inducing severe infection in sheep, which was serologically confirmed.

Concerning the clinical observations in the experimentally infected rams, the animals developed clinical signs (fever, increased respiratory rate, anorexia, and apathy) as soon as day four p.i., which is in accordance with previous experimental studies [7,21,22]. A febrile response around three to five days p.i. of sheep with *T. gondii* is a very consistent clinical finding as has been reviewed by Dubey [1]. Moreover, it has been demonstrated that the time of the febrile response depends on the administered dose of oocysts and the individuality of the animal [28,29]. Similarly, we have seen a late immunological response after the 2nd-week p.i. with an increase of the IgG titers of infected animals, which remained high from the 6th-week p.i. until the end of the experimental period.

The administration of sulphadimidine has been reported to reduce the abortion rate in naturally infected sheep and goat flocks [23,24]. It has also been reported that following toltrazuril treatment, the decrease of seropositivity rates in lambs indicates a significant reduction of new tissue cyst formation [30]. In the present study, the group that received a sulphadimidine treatment at two months p.i. (group C), had a similar immunological response with the animals that did not receive this treatment (group B) during the whole experiment. Maybe, the anatomical construction of the male reproductive system does not allow sulphadimidine to reach the testes. It is well known that the blood-testis barrier is one of the tightest blood-tissue barriers in mammalians, so only a few drugs with properties of a high degree of fat solubility and low binding to plasma proteins can pass through the blood-testis barrier [31]. A remarkable point of our study was that the administration of sulphadimidine 24 h p.i. (group D) resulted in a one-week delay of immune response compared to other infected groups, as well as in lower, but not significant, values of IgG up to the 8th week. Furthermore, the additional treatment of the second administration of sulphadimidine in week eight (group D) ensured no significantly lower values of IgG until the end of the experimental period compared to untreated or once treated animals (group B or C). The decrease in seropositivity rates indicates that treatment with sulphadimidine 24 h after the infection reduces the immune response of the *T. gondii* infected animals, linked to a potentially reduced parasite multiplication.

The genetic material of the parasite has been detected by PCR in the reproductive system of male goats, dogs, rams, bulls, and boars [5,6,7,32,33]. In the present study, parasite DNA was detected by PCR in three sperm samples, one in each of the infected groups (B, C, and D), 15 weeks p.i.; even though it does not discard the possibility that the parasitic agent was present in more samples. PCR is a highly specific test, but its sensitivity is limited [34]. A false-negative result in PCR could be due to a low dose of parasite inoculation, inadequate quantities of genetic material in the samples, loss of the genetic material during the extraction technique, or the different DNA sequences from *T. gondii* used for PCR [29,33,35]. Besides that, the negative results of PCR in 21 of the 24 infected animals 15 weeks p.i., could be attributed to that the excretion of the parasite through semen does not last for a long time or/and it is not constant. Moreover, epididymal sperm was used in this study because of the involvement of premature infected rams. *T. gondii* can be localized in the accessory genital glands with its DNA commonly found in seminal plasma [11]. Previous studies reported negative results in PCR but positive in bioassay or even both positive PCR and bioassay results, but not on the same day [5,6,7,32,33]. Furthermore, experimental trials should be carried out to investigate this aspect, with several time point checks in semen collection.

As much as possible, evaluated sperm parameters are needed to predict potential male fertility, while a combination of results is of higher predictable value [16]. In the present study, sperm quality parameters related to fertility potentials, such as concentration, viability, morphology, motility, and kinetics, were assessed [18,36]. Additionally, the functional activity of sperm cell membranes (HOST) and sperm DNA integrity (AOT) were estimated.

Information about the possible effect of *T. gondii* on the fertilization capacity of male hosts in animals is limited and contradictory. According to previous studies, *T. gondii* negatively affects the main sperm parameters in mice and rats [18,37,38]. De Moura et al. [20] did not find changes in basic semen quality parameters (volume, concentration, vitality, and motility) after infection with *T. gondii* tachyzoites, but they stated that a direct effect on spermatogenesis should be accompanied by a significant increase in the frequency of primary morphological abnormalities. Many researchers agree that another aspect of *T. gondii* infection is probably a direct or an indirect effect on hypothalamic-pituitary-gonadal (HPG) axis normal functionality that can cause hormonal disbalance with subsequent adverse effects on male reproductive ability [18,39,40,41,42].

We found significantly lower sperm viability in infected groups compared to the control group, while a positive correlation between ram sperm viability and fertility has been demonstrated [43,44,45,46]. On the other hand, the motility and the majority of the kinetic variables were not affected. CASA analysis was performed directly after epididymal sperm collection, while eosin-nigrosin staining took place a short time later according to the routine of laboratory analysis. A possible increased susceptibility of infected animals’ spermatozoa membranes under handling and thermal processes led to lower viability compared to motility values. Semen morphology and the relationship with fertility has also been confirmed as an important indicator of semen fertilizing capacity in different species [16,47,48]. Despite sulphadimidine treatment, the infected groups showed higher rates of head, tail, and total morphological abnormalities compared to the control group. However, the observed percentages of abnormalities are considered normal in ram semen. In agreement with our study, previous studies in rats and mice reported a significant increase of morphological abnormalities after *Toxoplasma* infection [18,37,38], while Lopes et al. [21] did not directly relate the high percentage of distal droplets in ram spermatozoa with the *Toxoplasma* infection. Moreover, it is known that abnormal sperm tails and droplets might influence semen velocity [49]. Hulet et al. [50] reported a significant correlation of motility and some morphological sperm abnormalities with sheep fecundity. Motility is the most important characteristic associated with the migration of sperm through the female reproductive tract to the oviduct region, affecting sperm fertilizing capacity. Even though progressive and total motility were not significantly affected, we can consider that infected rams are at a greater risk of presenting with lower fertilizing ability compared to non-infected because of the viability and morphology results.

The combination of motility parameters and velocity traits, measured by a computer-assisted sperm analyzer, provides objective information about semen quality and potential fertility [51,52,53]. However, in sheep, the literature data are contradictory, while a few reports achieved to correlate ram sperm kinematic properties, measured by CASA, to field fertility. Vicente–Fiel et al. [44] found that adult rams of high field fertility exhibit significantly higher values of VCL, VSL, VAP, LIN, STR, and ALH compared to rams of low field fertility but they performed a completely different approach from the present study. Although total motility of the control group was higher compared to infected groups, the obtained results of our study did not reveal any dramatic effect of the parasitism in sperm motility. Santolaria et al. [45] reported that VCL and sperm viability show significant predictive capacity on ram field fertility. Both abovementioned parameters were higher in the sperm samples of control animals in our experiments, with significant differences only in viability. Moreover, Robayo et al. [54] have demonstrated that VCL and VAP are positively correlating with the ability of ram sperm to migrate in homologous cervical mucus, which is a step of the fertilization process. In our study, no significant differences in VCL and VAP were noticed between groups. However, the higher values of VCL and VAP in the control group affected the parameters of LIN (VSL×100÷VCL) and STR (VSL×100÷VAP), which presented significantly lower LIN values in control compared to B and C infected groups and STR in control compared to group C. Furthermore, significantly higher values of BCF were found in the untreated infected B group compared to the treated infected C group. BCF is the number of times the sperm head crosses the direction of movement, developing another flagellar wave. This result, alone, cannot be appreciated as high value, because among CASA semen evaluated parameters, assessment in combination with progressive motility, ALH, BCF, and VSL are much more correlated with in vivo fertility than the estimation of single kinetic parameter [47]. Additionally, according to Herrara et al. [55] in vitro fertilization (IVF) is significantly correlated to progressive motility but not to BCF. Therefore, the results of the present study about the major kinetic characteristics of ram spermatozoa indicate a light disorder of *T. gondii* infected animals’ sperm kinetics, whose effects probably are not significant for fertility.

From the point of view of spermatology, an interesting major finding of our study was the significantly higher percentage of HOST-positive spermatozoa in control compared to all infected groups. To our knowledge, no other study has evaluated the effect of *T. gondii* on sperm membrane functional integrity. This result is a strong indication of the detrimental effect of *T. gondii* on ram spermatozoa under the conditions of the induced experimental infection. The integrity and functional activity of spermatozoa membranes are important for sperm metabolism and fertilization process because sperm capacitation, acrosome reaction, and sperm binding to zona pellucida require intact and active membranes. HOST evaluates whether a physically and functionally intact membrane is biochemically active and capable of maintaining equilibrium between the sperm cell and its environment. In ruminants, positive HOS-Test spermatozoa have a high correlation with viability, motility, normal morphology, and fertility [56,57].

To the best of our knowledge, no published study evaluated the effect of experimental ram infection with *T. gondii* on sperm nuclear chromatin integrity. DNA fragmentation has been correlated to fertility in rams [44] and has been associated with reproductive failure at the onset of embryonic DNA expression and pregnancy outcome [58,59]. In the present study, no differences were observed between groups, so *T. gondii* had no negative effect on spermatozoa nuclear chromatin integrity.

Success in detecting histological lesions or cysts in body tissues following experimental *Toxoplasma* infection depends on the infection oocyst dose, particularly in large animal species because of the low number of presented parasites [28]. In the present study, the histopathological findings correspond to sperm morphological abnormalities, which were found in the infected groups. Many years ago, the morphological abnormalities were classified as primary, secondary, or tertiary according to their origin. Primary morphological abnormalities, like the head defects, are created during spermatogenesis when the cells are still in the seminiferous epithelium of the testis. Secondary abnormalities arise after the sperm cells have left the testis at the epididymal passage and during the storage of spermatozoa, while tertiary sperm abnormalities arise from the improper handling of semen samples [60]. Higher values of relative to spermatogenesis and sperm migration morphological abnormalities were found in infected C and D groups of our study compared to the control group. It is one more indication that *T. gondii* infection disturbs the functionality of the testis and epididymis. Santana et al. [7] and Terpsidis et al. [18] did not observe significant histopathological changes related to *T. gondii* parasitism on male goats and mice reproductive systems, respectively. On the contrary, Dvorakova–Hortova et al. [42], after experimental *Toxoplasma* infection of mice, found a low number of leptotene primary spermatocytes and spermatids, higher number of Sertoli cells, and elevated tubule diameter histopathological changes.

Research data indicate the beneficial effect on infected female sheep of monensin [22] or sulphadimidine administration [23,24]. A similar beneficial effect is also reported for the administration of sulphamezathine and pyrimethamine [25]. In the present study, two different schedules of sulphadimidine treatment were performed in infected rams, while the effect on sperm quality traits was examined four months p.i. No beneficial results for sperm protection were obtained, making it necessary to carry out further experiments to clarify this issue.

## 4. Materials and Methods

### 4.1. Toxoplasma gondii Strain and Inoculation

The dose of 5 × 10^3^ sporulated *T. gondii* oocysts (76K strain, genotype II), in 5 mL of saline, was administered orally in each treated premature ram. Saline without oocysts (5 mL) was administered orally in each control premature ram.

### 4.2. Experimental Design, Animals

Healthy pre-pubertal 5 month old crossbred rams (*n* = 32) were selected. All animals were non-vaccinated and were serologically tested negative in *T. gondii* by an indirect commercial enzyme-linked immunosorbent assay ELISA kit test (IDEXX Toxotest Ab Test, IDEXX Europe B.V., Hoofddorp, The Netherlands). Subsequently, the animals were randomly assigned by age and nutritional status to four groups of eight individuals and housed in separate compartments protected from cats’ access. After a two week period of acclimatization, group A was confirmed as the control group (administration of saline), while the remaining three groups (B, C, and D) were orally infected with 5×10^3^ sporulated oocysts. In group B, no other treatment took place. Group C, received intramuscular (i.m.) sulphadimidine (Sulphadimidine^®^, CEVA) two months post-infection (p.i.) in a dose of 33 mg/kg, four times in total, once every 48 h (q48 h) [23,24]. In group D, the same dose of sulphadimidine was administrated twice, 24 h p.i. and two months later. Blood samples were collected every 15 days to detect IgG Abs (ELISA). The study lasted four months up to the sexual maturation of rams when all of them were euthanized. Epididymal sperm samples were collected after slaughtering and examined for the presence of the parasite by PCR molecular analysis. Moreover, semen analysis tests were performed to evaluate epididymal sperm quality, while testes were forwarded for histopathological examination.

### 4.3. Clinical Examination

Two days before the experimental infection and up to the end of the experiments, all animals were monitored and clinically checked for the appearance of clinical signs. The body temperature was measured every morning at 10:00.

### 4.4. Serological Analysis

For the first four weeks p.i., blood samples were collected weekly from the jugular vein in vacuum tubes without anticoagulant. Then, the blood sampling was continued bi-weekly until the date of the animals’ euthanasia. Vacuum tubes were transported to the laboratory, where serum was obtained by centrifugation at 1500 rpm for 10 min and stored at −20 °C until further analysis [61,62]. The presence of specific IgG *T. gondii* antibodies was detected by an indirect commercial enzyme-linked immunosorbent assay ELISA kit test (IDEXX Toxotest Ab Test, IDEXX Europe B.V., Hoofddorp, The Netherlands), of high sensitivity and specificity [63]. The test was performed according to the manufacturer’s instructions. Briefly, during incubation time on microplate wells coated with an antigen of *T. gondii*; an antigen-antibody immune complex was formed in case of antibodies exist in the serum. The results were calculated by a built-in computer ELISA automatic analyzer test with the use of special program software (Brio 2 Win 1.10, SEAC S.R.L. Firenze, Italy). The results were expressed as optical density (OD) values, while an antibody titer of 0.595 or above was considered positive.

### 4.5. Molecular Analysis

DNA was extracted from the sperm samples (volume 0.1 mL extracted from the cauda epididymis) of all experimental rams using a commercial tissue kit and according to the manufacturer’s instructions (Qiagen, Germantown, MD, USA). After extraction, all the DNA samples were subjected to a PCR using a pair of primers for the amplification of a specific *T. gondii* DNA fragment (B1 gene region) as described by Fuentes et al. [64] and Boughattas et al. [65]. Negative and positive controls were added to each set of PCRs. After the reaction, the PCR product was detected by electrophoresis in 2% agarose gel, stained with a 0.5 μg/mL ethidium bromide solution in water for 20 min, and observed under ultraviolet light.

### 4.6. Epididymal Sperm Samples Collection

It was selected to collect sperm from the epididymal cauda because the spermatozoa of this region are functionally mature, and their fertilizing ability is almost equivalent to that of ejaculated sperm [66,67]. Both testes and epididymides contained in scrotal sacs were immediately removed after slaughtering, placed into an isothermal box (15 °C), and transferred to the laboratory in about 2 h. At room temperature, about 20 °C, each epididymal cauda was revealed from the scrotum, detached from the respective testis, and weighed on a high precision scale for the evaluation of sperm concentration. The epididymal sperm was collected through the proximal end of the severed epididymis by infusion of 5 mL Phosphate Buffered Saline (PBS) under pressure from the aperture of the deferent duct [47,68]. The 10 mL blend of each pair of epididymides were centrifuged (1500 rpm; 10 min; 20 °C), and the sediment was diluted to a 1:2 ratio with Ovixcell^®^ ram semen extender (IMV Technologies, L’Aigle, France) and preserved in a water bath (37 °C) until the end of the evaluation. Time elapsed between animal slaughtering and epididymal sperm collection ranged from four to five hours; an adequate time interval for evaluating sperm quality parameters [69,70].

### 4.7. Sperm Evaluation

#### 4.7.1. Concentration

Sperm concentration was measured by an improved Neubauer hemocytometer chamber (Marienfeld, Germany). Concentration was estimated as the number of spermatozoa ×10^6^/tissue gram of the pair of epididymides [18].

#### 4.7.2. Sperm Viability and Morphology Assessment

**Figure 6:**
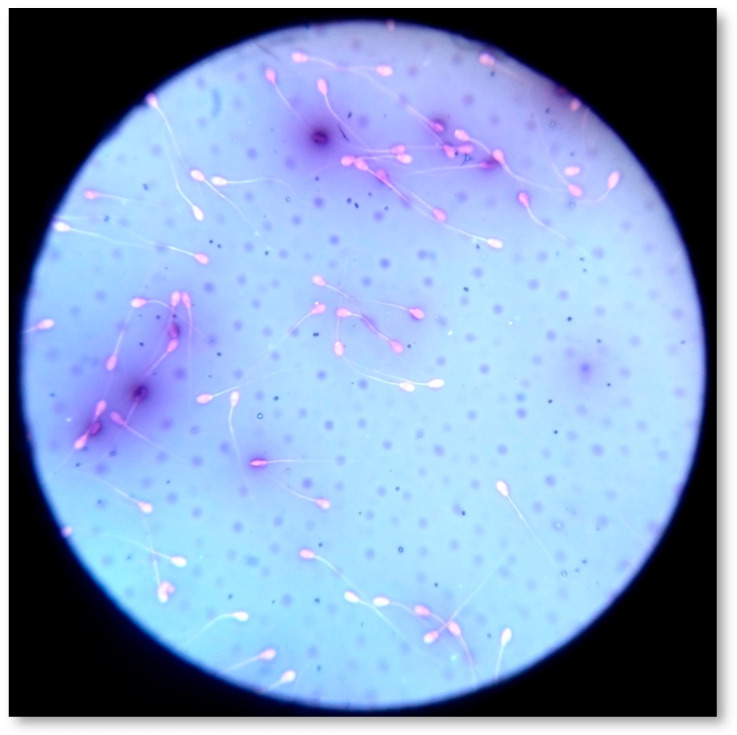
Estimation of sperm morphology and viability by one step Eosin-Nigrosin double stain.

Sperm viability and morphology were assessed by one step Eosin-Nigrosin (Sigma Aldrich^®^, Seelze, Germany) double stain method (Figure 6) [18,36,49]. Spermatozoa (*n* = 300) were counted by means of an optical microscope (AXIOSTAR Plus, ZEISS, Germany; magnification ×1000). The recorded live and dead spermatozoa were expressed in a percentage ratio (%). Concerning morphology, the abnormal spermatozoa were classified as follows: (1) abnormal head (detached head, defects in size and shape); (2) midpiece defects (distal midpiece reflex, bowed midpiece, and others); (3) tail defects (bent tail, coiled tail, and others); and (4) presence of proximal cytoplasmic droplets [71]. If multiple abnormalities were identified on individual spermatozoa, all were recorded; thus, the actual frequency of each abnormality in the population was determined. In accordance with the major or minor impact of morphological abnormalities on male fertility [72], the total percentage (%) of abnormal spermatozoa was estimated by counting once those who had more than one abnormality, using the following priority: abnormal head, midpiece, tail, presence of droplets. Spermatozoa (*n* = 300) were scored at magnification ×1000, and the % ratio was calculated for each category.

#### 4.7.3. Computer-Assisted Sperm Motility and Kinetics Analysis

The sperm samples were diluted before analysis to a final concentration of 20 – 25 × 10^6^ spermatozoa/mL. For CASA analysis (Sperm Class Analyzer^®^ Microptic SL, Barcelona, Spain) purposes, an aliquot of 10 μL sperm sample was placed on a warmed (37 °C) Makler^®^ counting chamber (10 μm deep, Sefi Medical Instruments, Haifa, Israel), and pictures were taken (×100; AXIO Scope A1 Optical microscope, Zeiss, Oberkochen, Germany), to record the movements of at least 500 spermatozoa. Total motility (%), progressive motile, rapid, medium, slow, and immotile spermatozoa, as well as the values of curvilinear velocity (VCL) (μm/s), straight-line velocity (VSL) (μm/s), average path velocity (VAP) (μm/s), linearity (LIN) (VSL×100÷VCL), amplitude of lateral head displacement (ALH) (μm), straightness (STR) (VSL×100÷VAP), beat cross-frequency (BCF) (Hz), and hyperactive spermatozoa (%), were assessed.

CASA software was configured as follows: 10 fields and >500 spermatozoa, 50 frames/s, region of particle control: 3–70 μm^2^, progressive movement >80% of the parameter STR, circumferential movement <50% of LIN, depth of field 10 μm and temperature of the microscope plate at 37 °C.

The incorrectly identified as spermatozoa objects were manually removed. Each measurement was performed twice to achieve an acceptably low sampling error.

#### 4.7.4. Sperm Membrane Biochemical Functionality

**Figure 7:**
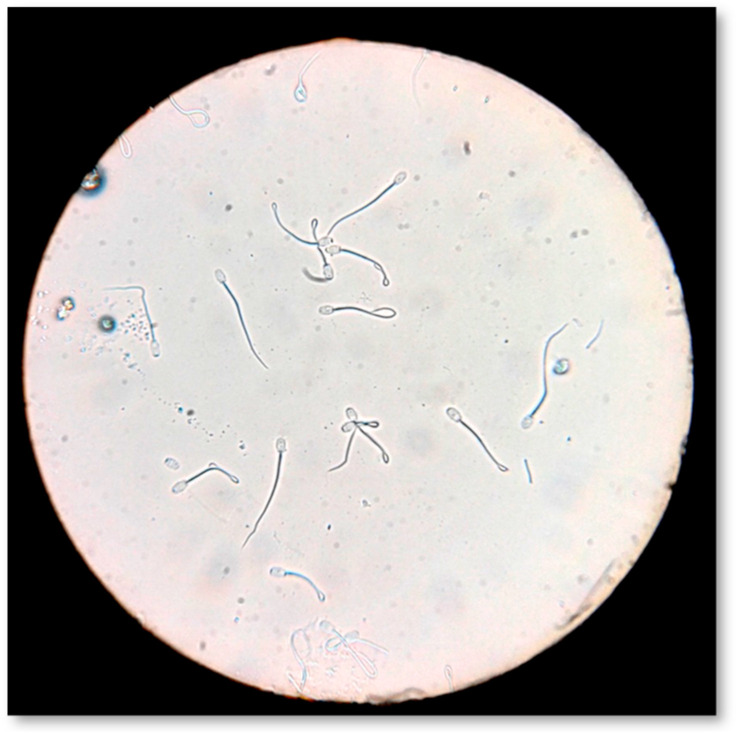
Evaluation of sperm membrane biochemical functionality by hypo-osmotic swelling test (HOS-Test).

Sperm plasma membrane biochemical functionality was evaluated by the hypo-osmotic swelling test (Figure 7), according to Pelufo et al. [73]. Briefly, 0.9 mL of hypo-osmotic solution (150 mOsm/L) and 0.1 mL of sperm were incubated at 37 °C for 30 min. After incubation, 20 μL of the sample was spread on a slide. Under light microscopy (AXIOSTAR Plus, Optical microscope, ZEISS, Germany; magnification ×400), 300 spermatozoa were counted and classified as HOST-positive (coiled or swollen tails) and HOST-negative (non-coiled or no-swollen tails). The results were expressed as the percentage (%) of HOST-positive spermatozoa.

#### 4.7.5. Sperm DNA Integrity

**Figure 8:**
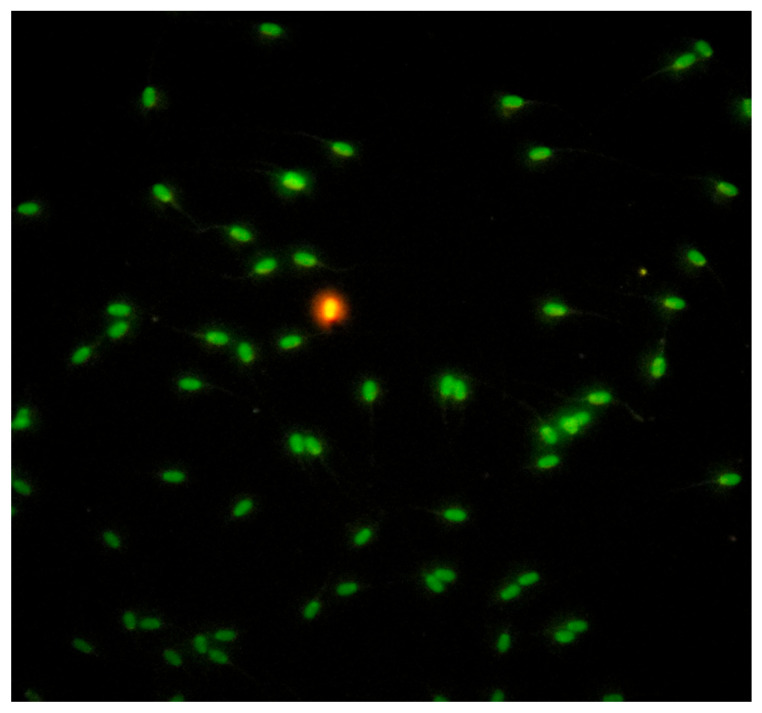
Evaluation of sperm chromatin integrity by Acridine Orange Test (AOT).

Sperm nuclear chromatin integrity evaluated by the Acridine Orange Test (AOT), (Sigma Aldrich^®^, Seelze, Germany) [74]. The test estimates the susceptibility of spermatozoa nuclear DNA integrity by altering the color of the fluorochrome acridine orange from green (normal double-stranded DNA) to red (denatured single-stranded DNA), under fluorescence light microscopy (fluorescence microscope AXIO, Scope A1, ZEISS, Germany), (Figure 8). A total of 200 spermatozoa per sample were estimated and counted. Spermatozoa with head colored from orange to red tint considered to be DNA damaged, while the results were expressed in percentage ratio (%).

### 4.8. Histopathological Examination

All experimental and control animals were examined histopathologically. Multiple tissue samples of 0.5 cm thick were collected from the left and right testicles and epididymides and fixed in 10% neutral buffered formalin for approximately 24 h. The samples were processed routinely and embedded in paraffin wax. 4–5 μm tissue was cut and stained with hematoxylin and eosin (HE) for examination under an optical microscope.

### 4.9. Statistical Analysis

Both parametric and nonparametric statistical methods were applied for the statistical evaluation of the results. Data were reported as mean ± standard deviation. A mixed repeated-measures analysis of variance (ANOVA) test was performed to determine the mains effects of *T. gondii* (between-subjects factor) and time (within-subjects factor) on serum IgG titers, as well as their interaction (*T. gondii* × time). The statistical assessment of sperm quality parameters between *T. gondii* groups (treatments) was performed through the application of one-way ANOVA. The assumptions of normality and homogeneity of variances were tested using the Shapiro–Wilk and Levene’s test, respectively. Differences between mean values of specific treatments were evaluated using posthoc multiple comparison tests (Tukey test). Where assumptions about either variability or the form of the population’s distribution were seriously violated, the Kruskal–Wallis non-parametric test was applied to evaluate treatment dependent differences, while differences between mean values of specific treatments were evaluated using the Wilcoxon rank-sum test (Mann–Whitney U-test). All analyses were performed using the statistical software program IBM SPSS (version 25.0 program, provided by the Aristotle University of Thessaloniki). The level of significance of the findings was determined at the level of 0.05.

### 4.10. Ethics Statement

The experimental protocol was approved by the Ethics Committee of “General Directorate of Regional Rural Economy and Veterinary, Region of Epirus—Greece”; approval code: 15405; date of approval: 26 October 2016. Housing and care of animals used in the scientific procedures were conducted in accordance with the Greek legislation (Presidential Decree No. 56, 2013, volume A, page 106). Transportation and slaughter of the animals conformed to European Regulations (EC) 1/2005 and (EC) 1099/2009, respectively.

## 5. Conclusions

The experimental infection of rams during the pre-pubertal period of age with oocysts of *T. gondii* degrades sperm quality at puberty, even four months after infection, while the administration of sulphadimidine could not prevent this effect.

## Figures and Tables

**Table 1:** Effect of blood sampling time on IgG titers.

Weeks of Blood Sampling	Optical Density Values—ELISA
0	0.09 ± 0.05 ^a,b^ (0.0008)
1	0.08 ± 0.03 ^a^ (0.007)
2	0.15 ± 0.09 ^b^ (0.018)
3	0.88 ± 0.70 ^c^ (0.085)
4	1.55 ± 1.07 ^d^ (0.085)
6	2.03 ± 1.18 ^e^ (0.075)
8	1.93 ± 1.24 ^d,e^ (0.129)
10	1.84 ± 1.12 ^d,e^ (0.070)
11	1.79 ± 1.06 ^d,e^ (0.070)
13	1.70 ± 1.09 ^d,e^ (0.091)
15	1.90 ± 1.16 ^d,e^ (0.097)

Values are mean ± standard deviation (SD). Numbers in parentheses are standard error of measurement (SEM). Different letter superscripts denote significant differences between weeks (*p* < 0.05). Optical density values with an antibody titer of 0.595 or above were considered positive. ELISA: enzyme-linked immunosorbent assay.

**Table 2:** Effect of *T. gondii* and blood sampling time on IgG titers.

Weeks of Blood Sampling	Optical Density Values—ELISA			
	Group A	Group B	Group C	Group D
0	0.08 ± 0.04 (0.017)	0.12 ± 0.06 (0.016)	0.08 ± 0.03 (0.016)	0.10 ± 0.04 (0.018)
1	0.07 ± 0.02 (0.013)	0.09 ± 0.06 (0.012)	0.08 ± 0.02 (0.012)	0.08 ± 0.02 (0.014)
2	0.15 ± 0.07 (0.037)	0.14 ± 0.09 (0.035)	0.17 ± 0.12 (0.035)	0.17 ± 0.10 (0.040)
3	0.12 ± 0.03 ^a^ (0.171)	1.25 ± 0.70 ^b^ (0.160)	1.46 ± 0.38 ^b^ (0.160)	0.52 ± 0.38 ^a^ (0.185)
4	0.08 ± 0.02 ^a^ (0.172)	2.12 ± 0.51 ^b^ (0.161)	2.57 ± 0.20 ^b^ (0.161)	1.14 ± 0.78 ^c^ (0.186)
6	0.13 ± 0.02 ^a^ (0.151)	2.77 ± 0.38 ^b,c^ (0.142)	2.84 ± 0.44 ^b^ (0.142)	2.17 ± 0.57 ^c^ (0.164)
8	0.10 ± 0.02 ^a^ (0.261)	2.42 ± 0.70 ^b^ (0.244)	2.69 ± 0.84 ^b^ (0.244)	2.42 ± 0.84 ^b^ (0.282)
10	0.07 ± 0.01 ^a^ (0.141)	2.69 ± 0.45 ^b^ (0.132)	2.52 ± 0.47 ^b^ (0.132)	1.87 ± 0.33 ^c^ (0.153)
11	0.12 ± 0.04 ^a^ (0.142)	2.55 ± 0.45 ^b^ (0.133)	2.50 ± 0.31 ^b^ (0.133)	1.78 ± 0.53 ^c^ (0.153)
13	0.09 ± 0.04 ^a^ (0.183)	2.42 ± 0.57 ^b^ (0.172)	2.51 ± 0.32 ^b^ (0.172)	1.52 ± 0.77 ^c^ (0.198)
15	0.18 ± 0.11 ^a^ (0.197)	2.56 ± 0.38 ^b,c^ (0.184)	2.79 ± 0.46 ^b^ (0.184)	1.87 ± 0.92 ^c^ (0.213)

Group A control, group B infected, group C infected and once treated with sulphadimidine, group D infected and twice treated with of sulphadimidine. Values are mean ± SD. Numbers in parentheses are standard error of measurement (SEM). Different letter superscripts denote significant differences between groups (*p* < 0.05). Optical density values with an antibody titer of 0.595 or above were considered positive.

**Table 3:** Effect of *T. gondii* on IgG titers results.

Group	Optical Density Values—ELISA
A	0.11 ± 0.05 ^a^ (0.047)
B	1.74 ± 1.15 ^b^ (0.044)
C	1.84 ± 1.18 ^b^ (0.044)
D	1.24 ± 1.00 ^c^ (0.050)

Group A control, group B infected, group C infected and once treated of sulphadimidine, group D infected and twice treated with of sulphadimidine. Values are mean ± SD. Numbers in parentheses are standard error of measurement (SEM). Different letter superscripts denote significant differences between groups (*p* < 0.05). Optical density values with an antibody titer of 0.595 or above were considered positive.

**Table 4:** Sperm quality parameters.

Sperm Quality Parameters	Group A	Group B	Group C	Group D
Concentration x10^6^/gr tissue	21.65 ± 6.73 (2.097)	15.90 ± 14.49 (5.121)	14.46 ± 7.10 (2.510)	15.48 ± 8.06 (3.193)
Viability (%)	75.71 ± 4.46 ^a^ (1.488)	42.28 ± 10.22 ^b^ (3.613)	41.33 ± 10.98 ^b^ (3.881)	48.55 ± 10.89 ^b^ (3.443)
Morphological abnormalities in total (%)	4.82 ± 2.35 ^a^ (0.783)	7.61 ± 3.93 ^a,c^ (1.390)	12.06 ± 3.19 ^b^ (1.127)	8.85 ± 2.51 ^b,c^ (0.793)
Head defects (%)	1.53 ± 1.18 ^a^ (0.394)	3.04 ± 3.25 ^a,b^ (1.150)	3.19 ± 1.28 ^b^ (0.453)	1.91 ± 0.77 ^a^ (0.243)
Midpiece defects (%)	0.45 ± 0.5 (0.167)	1.06 ± 1.10 (0.389)	1.48 ± 1.32 (0.468)	0.92 ± 0.79 (0.248)
Proximal cytoplasmic droplets (%)	1.50 ± 1.16 (0.388)	1.76 ± 1.31 (0.463)	3.23 ± 2.00 (0.710)	3.46 ± 1.91 (0.605)
Tail defects (%)	1.34 ± 1.01^a^ (0.337)	1.75 ± 1.03 ^a^ (0.363)	4.16 ± 1.99 ^b^ (0.703)	2.55 ± 0.87 ^a,b^ (0.275)
Host-positive spermatozoa (%)	70.48 ± 4.20 ^a^ (1.399)	39.38 ± 12.61 ^b^ (4.457)	51.91 ± 13.55 ^b^ (4.792)	47.84 ± 9.01 ^b^ (2.849)
DNA fragmentation (%)	0.09 ± 0.15 (0.048)	0.11 ± 0.22 (0.079)	0.54 ± 1.48 (0.523)	0.2 ± 0.32 (0.100)

Group A control; group B infected; group C infected and once treated with sulphadimidine; group D infected and twice treated with sulphadimidine. Values are mean ± SD. Numbers in parentheses are standard error of measurement (SEM). Different letter superscripts denote significant differences between groups (*p* < 0.05).

**Table 5:** Sperm motility and kinetics, CASA analysis.

Parameters	Group A	Group B	Group C	Group D
Progressive motile spermatozoa (%)	10.75 ± 2.06 (0.688)	14.42 ± 7.87 (2.783)	16.2 ± 7.83 (2.770)	14.8 ± 7.35 (2.325)
Total motility (%)	96.74 ± 3.82 (1.272)	80.80 ± 24.44 (8.642)	89.78 ± 12.92 (4.569)	91.35 ± 13.94 (4.407)
Rapid moving spermatozoa (%)	50.19 ± 27.39 (9.130)	38.81 ± 31.79 (11.240)	29.20 ± 26.66 (9.425)	46.31 ± 30.72 (9.715)
Medium moving spermatozoa (%)	21.02 ± 8.60 (2.868)	15.49 ± 7.26 (2.567)	24.19 ± 13.95 (4.932)	20.04 ± 9.21 (2.912)
Slow moving spermatozoa (%)	25.53 ± 17.34 (5.781)	26.49 ± 19.08 (6.744)	36.4 ± 15.79 (5.582)	25.00 ± 19.53 (6.177)
Immotile spermatozoa (%)	3.26 ± 3.82 (1.272)	19.20 ± 24.44 (8.642)	10.22 ± 12.92 (4.569)	8.65 ± 13.94 (4.408)
VCL (μm/s)	93.42 ± 47.78 (15.928)	75.36 ± 34.29 (12.123)	62.94 ± 25.94 (9.173)	81.44 ± 36.52 (11.547)
VSL (μm/s)	27.88 ± 12.20 (4.065)	28.10 ± 12.62 (4.463)	26.58 ± 10.43 (3.687)	28.56 ± 10.89 (3.443)
VAP (μm/s)	51.62 ± 25.10 (8.367)	44.50 ± 20.80 (7.355)	41.18 ± 16.44 (5.812)	48.24 ± 19.82 (6.267)
BCF (Hz)	10.43 ± 2.78 ^a,b^ (0.927)	15.22 ± 4.21 ^a^ (1.487)	9.90 ± 3.96 ^b^ (1.400)	11.36 ± 4.34 ^a,b^ (1.371)
ALH (μm)	2.72 ± 0.64 (0.213)	2.24 ± 0.39 (0.138)	2.41 ± 0.74 (0.262)	2.62 ± 0.71 (0.223)
LIN (VSL×100÷VCL)	30.71 ± 3.62 ^a^ (1.208)	37.89 ± 7.19 ^b^ (2.542)	43.13 ± 10.78 ^b^ (3.810)	35.86 ± 6.45 ^a,b^ (2.040)
STR (VSL×100÷VAP)	55.03 ± 4.85 ^a^ (1.616)	64.25 ± 9.58 ^a,b^ (3.389)	64.98 ± 7.92 ^b^ (2.801)	59.58 ± 6.83 ^a,b^ (2.160)
Hyperactived spermatozoa (%)	3.06 ± 1.67 (0.557)	3.39 ± 2.98 (1.053)	3.32 ± 3.51 (1.242)	3.81 ± 2.39 (0.756)

Group A control, group B infected, group C infected and once treated with sulphadimidine, group D infected and twice treated with sulphadimidine. CASA: Computer-Assisted Sperm Analysis; VCL: Curvilinear Velocity; VSL: Straight Line Velocity; VAP: Average Path Velocity; BCF: Beat-Cross Frequency; ALH: Amplitude of Lateral Head displacement; LIN: Linearity; STR: Straightness. Values are mean ± SD. Numbers in parentheses are standard error of measurement (SEM). Different letter superscripts denote significant differences between groups (*p* < 0.05).
